# A Mindfulness-Based App Intervention for Pregnant Women: Protocol for a Pilot Feasibility Study

**DOI:** 10.2196/53890

**Published:** 2024-05-10

**Authors:** Silvia Rizzi, Stefania Poggianella, Maria Chiara Pavesi, Lorenzo Gios, Giorgia Bincoletto, Isabella Scolari, Claudia Paoli, Debora Marroni, Irene Tassinari, Barbara Baietti, Anna Gianatti, Veronica Albertini, Barbara Burlon, Vanda Chiodega, Barbara Endrizzi, Elena Benini, Chiara Guella, Erik Gadotti, Stefano Forti, Fabrizio Taddei

**Affiliations:** 1 Digital Health Research Centre for Digital Health & Wellbeing Fondazione Bruno Kessler Trento Italy; 2 Istituto Pavoniano Artigianelli Trento Italy; 3 TrentinoSalute4.0 Competence Center for Digital Health Trento Italy; 4 Facoltà di Giurisprudenza Università degli Studi di Trento Trento Italy; 5 Operating Unit of Psychology Healthcare Trust of the Autonomous Province of Trento (APSS) Trento Italy; 6 Transmural Obstetric Gynecological Department Healthcare Trust of the Autonomous Province of Trento (APSS) Trento Italy

**Keywords:** mindfulness, promoting well-being, pregnancy, eHealth, mobile health, mHealth, mobile apps, development, usability, user-centered design, mindfulness based, intervention, pregnant women, pregnant, feasibility study, well-being, women, quality of life, psychological symptoms, digital solution, virtual coach, smartphone, mobile phone

## Abstract

**Background:**

Pregnancy is a complex time characterized by major transformations in a woman, which impact her physical, mental, and social well-being. How a woman adapts to these changes can affect her quality of life and psychological well-being. The literature indicates that pregnant women commonly experience psychological symptoms, with anxiety, stress, and depression being among the most frequent. Hence, promoting a healthy lifestyle focused on women’s psychological well-being is crucial. Recently developed digital solutions have assumed a crucial role in supporting psychological well-being in physiologically pregnant women. Therefore, the need becomes evident for the development and implementation of digital solutions, such as a virtual coach implemented in a smartphone, as a support for the psychological well-being of pregnant women who do not present psychological and psychiatric disorders.

**Objective:**

This study aims to assess the feasibility, acceptability, and utility of a mindfulness-based mobile app. The primary objective is to explore the feasibility of using a virtual coach, Maia, developed within the TreC Mamma app to promote women’s psychological well-being during pregnancy through a psychoeducational module based on mindfulness. Finally, through the delivery of this module, the level of psychological well-being will be explored as a secondary objective.

**Methods:**

This is a proof-of-concept study in which a small sample (N=50) is sufficient to achieve the intended purposes. Recruitment will occur within the group of pregnant women belonging to the pregnancy care services of the Trento Azienda Provinciale per i Servizi Sanitari di Trento. The convenience sampling method will be used. Maia will interact with the participating women for 8 weeks, starting from weeks 24 and 26 of pregnancy. Specifically, there will be 2 sessions per week, which the woman can choose, to allow more flexibility toward her needs.

**Results:**

The psychoeducational pathway is expected to lead to significant results in terms of usability and engagement in women’s interactions with Maia. Furthermore, it is anticipated that there will be improvements in psychological well-being and overall quality of life. The analysis of the data collected in this study will be mainly descriptive, orientated toward assessing the achievement of the study objectives.

**Conclusions:**

Literature has shown that women preferred web-based support during the perinatal period, suggesting that implementing digital interventions can overcome barriers to social stigma and asking for help. Maia can be a valuable resource for regular psychoeducational support for women during pregnancy.

**International Registered Report Identifier (IRRID):**

RR1-10.2196/53890

## Introduction

### Overview

The transition to motherhood represents a profound shift in a woman’s life, necessitating a reorganization of her identity, skills, attitudes, and values [1]. Pregnancy is characterized by major transformations. Pregnancy brings about substantial changes that impact a woman’s physical, mental, and social well-being. How a woman adapts to these changes can affect her quality of life (QoL) and psychological well-being. The literature indicates that pregnant women commonly experience psychological symptoms, with anxiety, stress, and depression being among the most frequent. Specifically, between 12.2% and 39% of pregnant women are reported to experience symptoms of anxiety and psychosocial stress [2], the latter defined as “an imbalance that a pregnant woman perceives when she is unable to cope with the demands of the new condition, which manifests itself behaviourally and psychologically” [3]. These findings were also confirmed by the study conducted by Woods et al [4], which revealed that 6% of women reported high levels of stress symptoms, 78% exhibited low to medium stress symptoms, and 16% reported no stress symptoms. Other studies have investigated perinatal depression in pregnant women, which has been recognized as a prevalent clinical condition [5]. Specifically, the study [5] found an incidence of depressive symptoms reaching 14.5% during pregnancy and 49% during the first year after delivery. Therefore, both pregnancy and the transition to motherhood are commonly seen as vulnerable periods marked by escalating worries and fears about managing the changes ahead, including caring for the baby or children yet to be born [6]. Therefore, it is crucial that pregnancy is experienced positively by women, thereby fostering the development—spontaneous or voluntary—of a positive level of cognitive motivation [7]. Indeed, promoting a healthy lifestyle focused on the woman’s psychological well-being is central to her mental health and future relationship with the child or children.

To date, psychoeducational interventions promoting women’s psychological well-being during pregnancy are scarce. They tend to focus on the effect of such interventions on specific subgroups of pregnant women with psychiatric symptoms (eg, perinatal depression disorder). Using digital tools to promote psychological well-being can be optimal for pregnant women, who can benefit from the intervention by accessing them anywhere and at any time. In addition, digital interventions are designed to be flexible to the mother’s needs and reduce the barriers related to social stigma and help-seeking behaviors [8,9]. Therefore, promoting tailored and easily accessible digital interventions aimed at addressing the specific needs of mothers during the transitional period is crucial for fostering positive relationships with their children. In this regard, a growing body of evidence reports promising results concerning the effectiveness of evidence-based mindfulness interventions delivered via web for promoting the psychological well-being of pregnant women [10,11]. Studies have also found that the psychological state of the pregnant woman appears to be closely related to the course of the pregnancy and the psychological and cognitive development of the child. Indeed, the literature reports a positive association among anxiety, stress symptoms, the likelihood of preterm delivery, and the variability in fetal heart rate [12]. Therefore, mindfulness interventions provide an active approach to managing stress levels, anxiety symptoms, and depression, aiming to reduce them. Thus, developing skills related to this practice enables women to mindfully deal with many potentially critical situations, including the moment of childbirth.

In conclusion, the psychoeducational interventions heralded through the use of digital solutions reported so far are shown to be a viable solution with the potential to promote psychological well-being, support the psychological adaptation of women in physiological pregnancy, and prevent the development of clinically relevant symptoms.

### Local Background

The autonomous province of Trento (PAT) is committed to promoting community welfare by providing a comprehensive initiative supporting and empowering parents at the provincial level. This includes a wide range of preventive, protective, and curative interventions spanning from early pregnancy to the first thousand days of a child’s life. These interventions are designed to have significant short-, medium-, and long-term health impacts for the newborn in both childhood and adulthood as well as for parents, the community, and future generations. In these initiatives, the integration of digital health tools is a crucial component within the framework of a pervasive digital health approach [13].

The overall strategy to support pregnant women begins with the active provision of the Birth Pathway initiative. The Birth Pathway encompasses all services aimed at promoting the health of women, infants or newborns, and families, providing appropriate care throughout pregnancy, childbirth, and postpartum, including support during breastfeeding, the postpartum period, and parenting.

Because of these comprehensive efforts at the provincial level, in 2022, the PAT and the provincial health services institution (Azienda Provinciale per i Servizi Sanitari—Azienda Provinciale per i Servizi Sanitari di Trento; APSS) achieved the United Nations International Children's Emergency Fund—Baby Friendly Initiatives accreditation for all birthing facilities and the community. This accreditation reflects the long-lasting commitment to the continuous improvement of care standards and outcomes. The PAT is also designated as Friends of Children, a status which the Trento birthing facility had already attained in 2015.

This research project has been developed in full harmonization with the overall principles of the PAT initiatives mentioned in the previous paragraphs and considering the broader provincial context in terms of organizational assets and service delivery. In particular, the project is promoted in the context of the First Thousand Days Module scenario of the Digital health care and artificial intelligence—tools for bringing the health service closer to citizens and for the development of the provincial system project, described and approved in deliberation number 2475 of the PAT on December 22, 2022. This strategy scenario is geared toward enhancing well-being and promoting healthy lifestyles during normal pregnancies, while also incorporating digital health principles and tools into organizational assets.

### Goal of the Study and Research Questions

This study is positioned within the design and development cycle of the Obesity-Related Behavioral Intervention Trials model (specifically the Refine phase) [14]. It involves delivering mindfulness-based materials in text, audio, and video formats by a virtual coach (ie, a digital assistant, hereafter identified as Maia) implemented within the TreC Mamma app.

This intervention is positioned within the framework of behavior change interventions [15], which aim to enable women to acquire adaptive strategies for their psychological well-being. It is emphasized that the term intervention in this context is used to identify an app (in this case, the virtual coach) that will periodically offer, in a predefined (ie, rule based), structured, and ordered manner, informational materials in audio, text, and video formats, which will be structured and reviewed by psychological professionals.

The end user reads and manages this material independently; therefore, the virtual coach will be used as a simple information transmission tool. In addition, the software (app and virtual coach) is used to administer questionnaires and collect, store, and transmit data in this project. This intervention has been developed and implemented for research purposes, with the final goal of validating a digital health intervention targeting women’s psychological well-being during pregnancy through a module of mindfulness practices.

### Primary Objectives

The primary objectives of this proof-of-concept study are (1) to exploratively investigate user experience (UX) and user engagement (UE), that is, women’s experience and engagement with the TreC Mamma app and the virtual coach dedicated to the mindfulness intervention (Maia); and (2) to assess the women’s UE with Maia and the TreC-Mamma app through semistructured interviews and investigate their feelings and overall experience during the psychoeducational intervention.

### Secondary Objective

The secondary objective is to assess the level of pre-post psychological well-being through administering self-report questionnaires at the beginning and the end of the intervention.

### Maia Intervention

The virtual coach, Maia, is structured to deliver a module featuring psychoeducational content based on mindfulness to promote the QoL and psychological well-being perceived by pregnant women. The module is self-administered and available on both Android and iOS devices. All the contents of the dialogues have been developed by a group of researchers from the Digital Health Research of the Bruno Kessler Foundation (Fondazione Bruno Kessler–Trento; FBK) of Trento and psychologists from the Istituto Pavoniano Artigianelli with specific skills in mindfulness protocols and communication. Subsequently, materials have been revised by a psychologist from the psychology operating unit of the APSS of Trento. The dialogues, videos, and audio tracks were developed based on an educational rather than an emergency management approach.

### Technological Tools

The technological component of the study is based on TreC Mamma. This platform allows citizens of the PAT to access, manage, and share information regarding their health and well-being [16]. TreC stands for *cartella clinica del cittadino* and is a reliable and well-tested platform designed to be a “system of systems” rather than a simple data hub. The central pillar of the TreC Mamma platform is the role of the citizen or patient as the manager of their own health-related data as in the case of a personal health record. TreC Mamma is designed with a flexible architecture, which enables the collection and management of heterogeneous data and allows the development and use of further subsystems to provide additional and specific functions.

This research project uses an additional module of the TreC Mamma platform, called TreC Mamma, a technological platform for supporting pregnant women who are residents in the PAT and are enrolled to APSS’s services. TreC Mamma can be downloaded for free by all pregnant women in the provincial health service. Authentication should be carried out via the *sistema pubblico di identità digitale* (ie, the public digital identity system) or *carta di identità elettronica* (ie, electronic identity card), which are secure national identification system services.

### Mindfulness Practices Module

**Figure 1:**
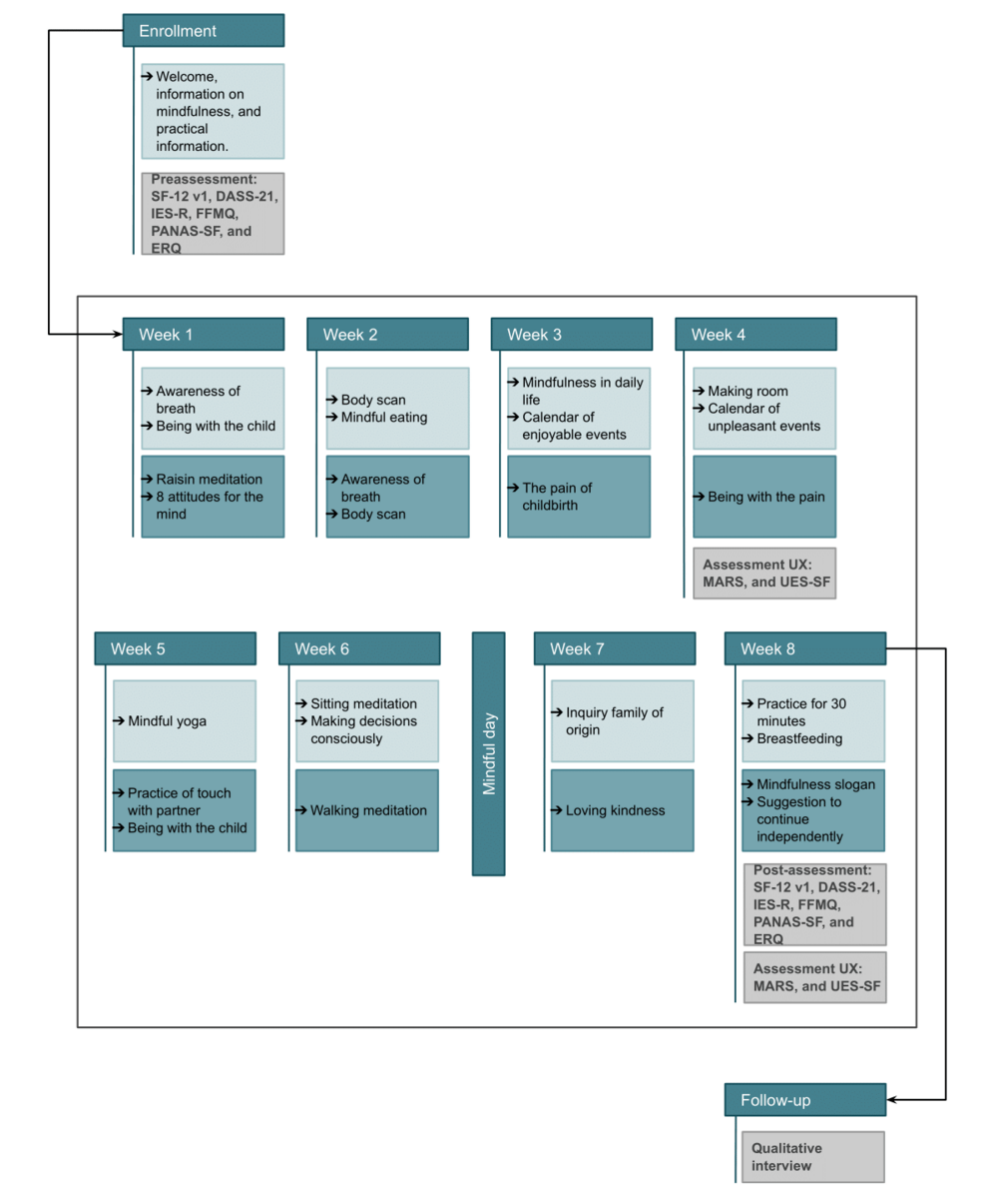
Graphical representation of the conversational protocol delivered to the participants and its chronological structure. DASS-2: Depression Anxiety Stress Scales–21; ERQ: Emotion Regulation Questionnaire; FFMQ: Five Facet Mindfulness Questionnaire; IES-R: Impact Event Scale–Revised; MARS: Mobile Application Rating Scale; PANAS-SF: Positive and Negative Affect Schedule–Short Form; SF-12v1: Short Form–12 Health Survey Version; UES-SF: User Engagement Scale–Short Form; UX: user experience.

The mindfulness practice intervention lasts 8 weeks, with 2 sessions of approximately 10 minutes each per week. Figure 1 shows a graphical representation of the conversational protocol delivered to women and its chronological structure.

This module aims to enable women to acquire healthy coping strategies with specific reference to mindfulness practices to promote psychological well-being during and after pregnancy. Pregnant women can learn mindfulness techniques to manage the typical anxiety and stress experienced during pregnancy, cope with pain and fear during childbirth, and enhance couple cooperation and parental sensitivity.

The module was developed in line with the mindfulness-based childbirth and parenting approach [17], adapting it to a psychoeducational intervention delivered via a virtual coach. The contents are presented through a gradual pathway that will enable the woman to be mindful of herself and the present moment, to accept and normalize internal states, to cope with stress, and to perceive an improvement in her psychological well-being. Specifically, formal practices (eg, “body scan”) and informal practices (eg, audio tracks on “eating a meal with mindfulness”), readings, or videos related to important topics about mindfulness practice will be proposed. Furthermore, the woman will be asked to carry out tasks independently. If she wishes, she can ask for support from her partner or a trusted person for some practices, but it is not mandatory.

## Methods

### Design and Study Plan

Maia is designed to interact with pregnant women 8 weeks from the 24th and 26th week of pregnancy. Precisely, 2 sessions per week are planned. The woman can choose the day and time that best suits her needs to interact with Maia.

At the beginning of the psychoeducational pathway, Maia will administer 6 self-report questionnaires, described in the Data Collection section, to establish a baseline.

After the data collection phase, the psychoeducational module will be administered, and its contents will be presented in different formats (ie, text, images, audio tracks, and video tracks).

An additional round of administering questionnaires is expected at the end of the psychoeducational intervention to assess potential changes in terms of the psychological well-being and QoL perceived by the women. Two questionnaires will be administered to assess usability. Reminders will be scheduled to fill out the questionnaires 24, 48, and 72 hours after the first request to ensure the collection of all the necessary data. However, at present, there are no reminders for session completion.

Two months after giving birth, women who have given their consent will be invited to a semistructured interview to investigate their experience of using Maia and the TreC Mamma app and to collect a more in-depth understanding of their experiences during the psychoeducational process. The 2-month postpartum period was chosen for both organizational convenience and methodological considerations. The expert group identified this timeframe to find a balance between scientific appropriateness and practical arrangements of the interviews, avoiding a prolonged span after the end of the intervention while ensuring that interviews with the patients do not occur too closely to the childbirth weeks. The underlying assumption is that the period immediately following childbirth (ie, the first 2 months) may be a logistically and emotionally complex time for the family, with a particular focus on the mother’s availability and experience.

### Participant Recruitment and Withdrawal

The study’s target population includes all pregnant women residents in the PAT.

The inclusion criteria to participate in the study are as follows: (1) be pregnant; (2) be in a gestational state between the 24th and 26th week; (3) be aged ≥18 years; (4) have a smartphone with internet access, be able to download the app, and be able to use it; (5) be a resident of the PAT; and (6) know and understand the Italian language.

The exclusion criteria are as follows: (1) patients unable to provide informed consent, which is a prerequisite for participation in the study; (2) inadequate understanding of the Italian language; (3) substance addiction or in recovery for <1 year; (4) suicidal tendencies; (5) psychosis; (6) posttraumatic stress disorder; (7) depression or other psychiatric diagnoses; (8) social phobia; (9) women undergoing medically assisted procreation; and (10) positive obstetric history for late intrauterine fetal death and stillbirth, polyabortion, therapeutic abortion, or sudden infant death syndrome.

Recruited women who meet the inclusion criteria and give their consent to participate in the study will be enrolled in the research. All women who decide to take part in the study will be asked to sign an informed consent form at the time of enrollment after a careful explanation of the project; its aims; how the data will be collected, managed, and processed; the level of involvement required; and the duration of the research as well as any confidentiality issues. The woman will also be informed of the possibility of quitting the study at any time she wishes without any explanation and that this option would not impact the quality of care or interfere with her course of treatment.

The participant must provide informed consent freely, voluntarily, and in writing.

Participants will also receive a privacy policy statement regarding the data processing of their personal data during the research. They should provide their explicit consent to the data processing.

Copies of the study’s informed consent form and privacy consent form will be provided to the participant. This information will also be available in a dedicated section of the app.

**Figure 2:**
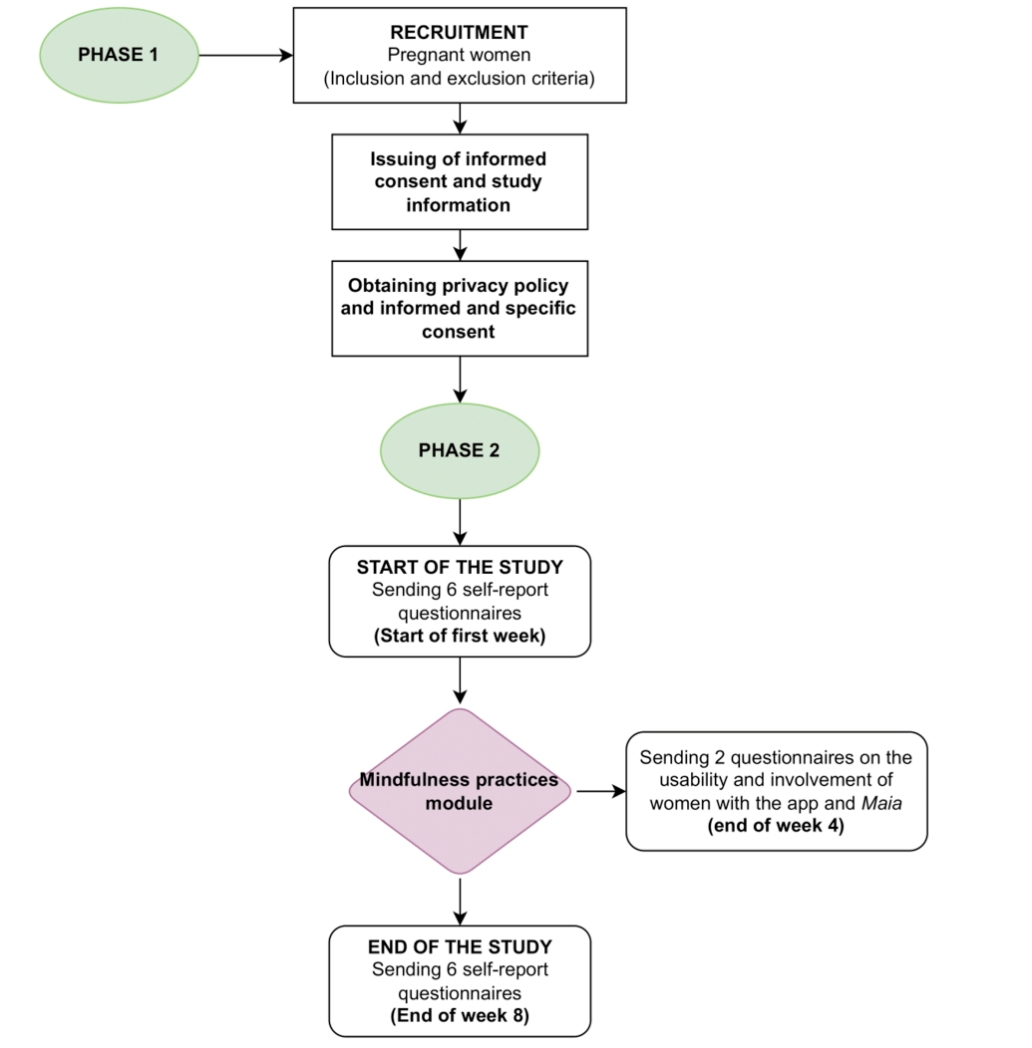
Flowchart of the research project procedure.

A flowchart of the research project procedure in sown in the Figure 2.

### Sample Size

This research project is designed as a proof-of-concept study, where a small sample is sufficient to achieve the intended aims. It was calculated that if nonparametric statistics are conducted (assuming the distribution is not normal), the sample should consist of 24 pregnant women with Bonferroni correction (Cronbach α=.025). The sample size and power for nonparametric tests (Kruskal-Wallis test and Wilcoxon post hoc test) c according to Noether [18]. If parametric statistics are conducted (assuming the normal distribution), the sample must instead consist of 41 pregnant women with Bonferroni correction (α=.025). Thus, with a power level set at 0.80, a significance level of .025, and factoring in a 20% potential dropout rate, we estimated that recruiting 50 women would be necessary to conduct the study.

### Role of Health Care Personnel

Pregnant women attending the pregnancy care services of the APSS will be recruited between the 24th and 26th week of gestation. To identify women potentially eligible to participate in the study and collect informed consents and initial sociodemographic data, the involvement of some midwives or doctors belonging to the hospital and territorial service of the APSS is planned.

### Study Outcomes

#### Primary Outcome

The primary outcomes are UX and UE. They will be assessed using 2 questionnaires, namely, the Mobile Application Rating Scale (MARS) [19] and the User Engagement Scale–Short Form (UES-SF) [20], which will be administered at different time slots throughout the study, in particular, at the end of week 4 and at the end of the study (ie, week 8).

The end points that will be measured are as follows: a score of the individual items of the questionnaires (MARS and UES-SF); the average score of the questionnaires; and the difference between the average score values of the individual questionnaires at different survey times.

The evaluation of the experience of using the app and the virtual coach (Maia) and the use of the intervention itself will also be assessed through semistructured interviews, conducted 2 months after childbirth. In this case, qualitative data will be collected to enrich the information related to the primary outcome.

#### Secondary Outcome

The variable expressing the “psychological well-being” outcome will be assessed by administering self-report questionnaires at the beginning (ie, week 1) and end (ie, week 8) of the psychoeducational course (refer to Table 1 for a detailed overview of the questionnaire adopted). The end points that will be measured are as follows: a score of the individual items of the questionnaires; the average score of the questionnaires; and the difference between the average score values of the individual questionnaires in the 2 survey times.

**Table 1:** Summary of the questionnaires administered and their timing.

At the beginning of the study (week 1)	At the end of week 4	At the end of the study (week 8)	Follow-up (2 months after birth)
SF-12 v1^a^	N/A^b^	SF-12 v1	Qualitative interview
DASS-21^c^	N/A	DASS-21	N/A
IES-R^d^	N/A	IES-R	N/A
FFMQ^e^	N/A	FFMQ	N/A
PANAS-SF^f^	N/A	PANAS-SF	N/A
ERQ^g^	N/A	ERQ	N/A
N/A	MARS^h^	MARS	N/A
N/A	UES-SF^i^	UES-SF	N/A

^a^SF-12 v1: Short Form–12 Health Survey Version 1.

^b^N/A: not applicable.

^c^DASS−21: Depression Anxiety Stress Scales–21.

^d^IES-R: Impact Event Scale–Revised.

^e^FFMQ: Five Facet Mindfulness Questionnaire.

^f^PANAS-SF: Positive and Negative Affect Schedule–Short Form.

^g^ERQ: Emotion Regulation Questionnaire.

^h^MARS: Mobile Application Rating Scale.

^i^UES-SF: User Engagement Scale–Short Form.

The mindfulness-based childbirth and parenting protocol is aimed at creating a connection between expectant mothers and their bodies and fetuses. The literature highlights the growing interest in mindfulness-based interventions [21] due to their consistent effects, including reduced depressive [22], anxiety [23], and stress symptoms [24] as well as enhanced emotional regulation [25] and sleep quality [26].

Therefore, we intend to incorporate variables such as maternal stress levels, frequency of mindfulness practice, and gestational age as predictors. Particularly, we will explore stress and anxiety (Impact Event Scale–Revised and Short Form–12 Health Survey, Version 1), depression (Depression Anxiety Stress Scales–21), emotion regulation (Positive and Negative Affect Schedule–Short-Form and Emotion Regulation Questionnaire), and mindfulness level (Five Facet Mindfulness Questionnaire).

These confounding variables are pivotal as they could impact both the independent variables and outcomes, ensuring a comprehensive understanding of the association between mindfulness and maternal health outcomes. By addressing these factors, we aim to deliver a more robust analysis of the efficacy of mindfulness interventions during pregnancy.

### Data Collection

The midwives will collect the sociodemographic data at the time of enrollment using a prestructured form. A unique alphanumeric code will be used to link the different pieces of information with the project’s participants. The sociodemographic data collection form and the data collected during the study will be kept separately. In addition, data will be pseudonymized to ensure confidentiality.

The parameters requested from the women will be (1) age, (2) week of pregnancy, (3) weight and height, (4) socioeconomic level, (5) educational level, (6) occupation, (7) marital status, (8) number of pregnancies, and (9) current or previous experience of psychological support (to investigate and describe the prevalence of women who have requested support to care for their psychological well-being).

The participants will also be asked if they would like to participate in a qualitative interview 2 months after delivery. In case of positive feedback, their telephone number will be collected to facilitate future contact and follow-up interviews.

To properly assess participants’ experience during the psychoeducational pathway, some questionnaires were adopted and delivered at the beginning, during, and at the end of the interaction with Maia (Table 1).

Specifically, 8 questionnaires will be administered at the beginning and the end of the psychoeducational intervention. Two questionnaires will be delivered during the psychoeducational pathway to assess the usability and engagement of the woman with the app and Maia. Specifically, the usability questionnaires will be administered at the end of week 4 and week 8 (ie, the end of the study).

A detailed description of each instrument that will be administered is presented in the following paragraphs.

Short Form–12 Health Survey Version 1 [27] is a self-report questionnaire characterized by 12 items assessed on different scales (Likert and dichotomous). The questionnaire investigates the QoL in 8 health dimensions to assess physical and mental health. Domains related to physical health include (1) general health, (2) physical functioning, (3) physical role, and (4) body pain. The mental health scales include (1) vitality, (2) social functioning, (3) emotional role, and (4) mental health. The questionnaire was translated and culturally adapted into various European languages and countries, including Italy, as part of the International Quality of Life Assessment project.

The Depression Anxiety Stress Scales–21 [28] is a self-report questionnaire consisting of 21 items based on a 4-point Likert scale, ranging from 0 (it never happened to me) to 3 (it almost always happened to me). This questionnaire is characterized by 3 scales investigating symptoms of anxiety, depression, and stress, including a total distress scale. Higher scores correspond to a higher level of distress. This scale was translated into Italian and validated in an Italian context [28]. The total scale found very good internal reliability (α=.90).

The Impact Event Scale–Revised [29] is a self-report questionnaire consisting of 22 items rated on a 5-point Likert scale, ranging from 0 (not at all) to 4 (extremely). This questionnaire assesses the impact of events that may be perceived as stressful through three scales: (1) intrusion, (2) avoidance, and (3) hyperarousal. This questionnaire will be administered in this research project to consider the investigated data net of the participant’s pregnancy situation, eliminating potential external stressors (eg, COVID-19). The questionnaire was translated and validated in an Italian context [29]. The 3 scales show good internal reliability: α=.78 for intrusion; α=.72 for avoidance; and α=.83 for hyperarousal.

The Five Facet Mindfulness Questionnaire [30] is a self-report questionnaire consisting of 39 items rated on a 5-point Likert scale, ranging from 1 (never or very rarely true) to 4 (very often or always true). The questionnaire assesses the tendency to be attentive in daily life using five scales: (1) observe, (2) describe, (3) act with awareness, (4) do not judge, and (5) do not react. Higher scores indicate greater awareness. The questionnaire was translated and validated in an Italian context [31]. The Cronbach α values were .74 for observation, .92 for description, .85 for acting with awareness, .91 for nonjudgment, .79 for nonreactivity, and .84 for total score, indicating good reliability.

The Positive and Negative Affect Schedule–Short Form [32] is one of the most widely used instruments to assess positive and negative affective states. It measures 2 distinct and independent dimensions: positive affection (PA) and negative affection (NA). The questionnaire consists of 20 adjectives, 10 for the PA subscale and 10 for the NA subscale. The PA subscale reflects the degree to which a person feels enthusiastic, active, and determined; the NA subscale refers to general unpleasant states, such as anger, guilt, and fear. The participant is asked to rate how they generally feel in the manner described by the adjective, responding on a 5-point Likert scale (1=not at all to 5=very much). The instrument has excellent psychometric properties: the internal consistency coefficient of the PA subscale ranges from 0.86 to 0.90 and that of the NA subscale ranges from 0.84 to 0.87. Furthermore, the test has good convergent and divergent validity. The 2 subscales show a low correlation (−0.12 to −0.23), and this characteristic aligns with the theory that the 2 factors, PA and NA, are independent. The questionnaire has been translated into many languages; the Italian version was validated by Terracciano et al [33] on a sample of 600 participants and replicated the psychometric characteristics of the original study.

The Emotion Regulation Questionnaire [34] is a 10-item scale designed to measure the tendency to use 2 emotional regulation strategies, that is, cognitive reappraisal and expressive suppression. Respondents answer each item on a 7-point Likert-type scale, ranging from 1 (strongly disagree) to 7 (strongly agree). Balzarotti et al [35] translated and validated the questionnaire in Italian, showing good psychometric characteristics. In this study, Cronbach α was .81 for cognitive reappraisal and .84 for expressive suppression.

The MARS [36] is characterized by 23 items rated on a 5-point Likert scale, ranging from 1 (poor) to 5 (excellent), which assesses the quality of the app and its functionalities (eg, virtual coach) on 4 dimensions of objective quality: engagement (5 items), functionality (4 items), esthetics (3 items), and information (7 items), as well as a final scale assessing subjective quality (4 items). The average of the scores of the individual items will be calculated to obtain an average quality score for each dimension investigated; subsequently, the average of the scores of the 4 objective quality dimensions will be calculated to obtain a total MARS score. The questionnaire also includes an “application-specific” section (6 items) to assess the potential impact of a particular app on the users’ knowledge, intentions, and so on. The total and subscale scores of the MARS have very high Cronbach α coefficients (.90 and .80-.89, respectively). The scale has been validated in an Italian context [36]. For this study, only the subscales “information,” “subjective quality of the app,” and “app-specific” will be considered for 17 items.

The UES-SF [37] is a short self-report questionnaire to assess UE with a digital solution. This measure includes 12 items based on a 5-point Likert scale, ranging from 1 (strongly disagree) to 5 (strongly agree). The questionnaire consists of four factors: (1) focused attention, which indicates the feeling of being immersed in the interaction; (2) perceived usability, which is the negative effect experienced due to the interaction and the effort expended; (3) esthetic attractiveness, which represents the graphical and visual appeal to a digital solution; and (4) the reinforcement factor (ie, reward). The reinforcement factor is a single factor that includes duration, which evaluates the overall success of the interaction; novelty, which examines the general interest related to the interaction with a digital solution; and, finally, the perceived engagement factor, which evaluates the overall enjoyment of the interaction. This questionnaire had not been translated into Italian and was therefore translated through the back translation procedure. The 4 scales show good internal reliability: ω=0.82 for focused attention, ω=0.86 for perceived usability, ω=0.84 for esthetic attraction, and ω=0.81 for reinforcement.

### Qualitative Interview

The interviews will be conducted by a psychologist from FBK. Interviews are structured with ad hoc items to purposively capture specific characteristics of the study and participants’ experiences. During the qualitative interview, the participants will also be asked if someone supported them during the course, whether it was helpful, and how easy it was to involve another person in some of the exercises. This will allow them to reason, in terms of experience, about whether to involve their partner or a trusted person in a more structured way in the intervention itself. Interviews will be conducted 2 months after the birth; will last approximately 20 minutes; and, subject to the woman’s consent, will be audio recorded to allow subsequent analysis.

The platform adopted for the intervention allows for the recording of activity logs, enabling proper mapping of the participants’ actions and experiences. This covers a range of logs, assessing whether the participant is properly exposed to the intervention protocol while recording use patterns (eg, session execution time, number of accesses, and use patterns of different features). Any missed sessions or interruptions will be considered as variables. These data will be correlated with acceptability scores during the analysis phase. In case a participant does not complete the activities and misses filling in specific questionnaires (leading to missing data), specific pieces of information on their experiences and platform use will be collected during the scheduled postintervention interview sessions.

### Privacy and Data Protection

Concerning data protection, a privacy policy will be formulated pursuant to articles 13 and 14 of the regulation EU 2016/679 of the General Data Protection Regulation, explaining the purposes and legal grounds of the data processing, how the personal data will be collected, the data collection categories, and how data will be managed, including the data retention period, the obligations of the data controllers, and the rights of the participants. The data controllers are APSS and FBK. Personal data are processed for specific research purposes within the scope of public interest tasks of the data controllers. Informed, voluntary, and explicit consent regarding the processing of particular categories of personal data (ie, data concerning health and data on self-reported behavioral habits in the area of lifestyle health) will be collected. Moreover, a specific consent will be asked on the possibility to contact the participant via telephone to carry out the interviews for the research.

All data collected will be kept confidential, managed by authorized persons, and data necessary for evaluating the study objectives will be pseudoanonymized before processing. Therefore, data are pseudonymized and not anonymized.

Copies of the information on the study and the privacy policy will be issued to the participants and made available permanently in a dedicated section of the app.

The study manager undertakes the responsibility to produce a report on the research and to ensure that the data are reported responsibly and consistently. Personal data will neither be disclosed nor disseminated, except in anonymized or aggregated form for publishing purposes. The publication of data resulting from this study will take place independently of the results obtained. The transmission or dissemination of the data, by means of scientific publications or presentations at congresses, conferences, and seminars, will take place exclusively in anonymous form using only aggregated data that prevent any identification of the participants, even indirectly. This sharing is expected to happen within 1 year after the conclusion of the study.

As the participants use the TreC Mamma app, they will be able to give their consent to be contacted for other research initiatives, including initiatives aimed at the continuous improvement of the platform and the acceptability and usability of the app, the collection of data in aggregated form for epidemiological purposes, and promoting specific projects in the social health care field. For all research activities, as the consent should be specific, dedicated information will be provided, and an ad hoc explicit consent will be requested. Using an eHealth mobile app as a “technological intermediary” to enroll users in medical research projects creates an “alliance with the citizens” of the PAT for the proactive use of personal data in scientific studies.

### Data Analysis

Preliminary data analysis is scheduled for early June 2024, while the final results will be available at the end of the study (second half of 2024). The publication of the results will take place within 1 year after the conclusion of the study.

#### Quantitative Analysis

Statistical data processing will be conducted using the software R (version 4.0.0; R Foundation for Statistical Computing), SPSS Statistics (IBM Corp), and Stata17 (StataCorp LLC).

Categorical variables will be summarized by absolute and percentage frequency distributions and quantitative variables of appropriate centrality and variability indexes.

Descriptive analyses will be calculated both for psychological variables (QoL, anxiety, stress, depression, distress, the impact of external events, mindfulness, positive and negative affect, and emotional regulation) and usability (UX) and user involvement (UE) variables. These analyses will be performed on the variables analyzed at the beginning, during, and at the end of the interaction with Maia.

The relationships between variables will be analyzed mainly through ad hoc statistical tests, such as the chi-square test, Fisher exact test, 2-tailed *t* test for paired data or Wilcoxon nonparametric tests, and the sign test (based on an assessment of compliance with assumptions), to understand the differences between the beginning and the end of the course in the same study sample with respect to the variables under investigation.

Furthermore, univariate logistic models or multinomial regression models will be presented. Finally, to consider possible confounding factors, multiple regression models will be proposed, where the effects of the explanatory variables on the outcome variables will be adjusted for possible confounders.

For each analysis, statistical significance will be set at a *P* value of ≤.05.

#### Qualitative Analysis

With regard to the analysis of the semistructured interviews, a text mining approach [38] will be used to extract the answers that appear repeatedly from the interviews that will be conducted with respect to the women’s experience of interacting with Maia and with respect to how they felt during the psychoeducational process.

### Ethical Considerations

This study was approved by the ethics committee of the APSS (15742; September 14, 2023). At the time of enrollment, all women who freely decide to take part in the study will be asked to sign an informed consent form after receiving a careful explanation of the study, its aims, the level of involvement required, and the duration of the research, as well as all ethical issues concerning confidentiality (the explanation of the study will be carried out partly by the midwives, but a video recorded by the research team with an explanation of the study, its purpose, and the woman’s level of involvement in simple and understandable words will also be available). The participants will be informed of the study's results through the app.

## Results

The psychoeducational course delivered through a virtual coach, Maia, integrated within the TreC Mamma app, is expected to have significant results regarding women’s satisfaction and involvement with the innovative digital solution.

Moreover, an increase in the levels of psychological well-being, QoL of the pregnant woman, emotional regulation, and positive affect is expected at the end the intervention compared to the beginning.

## Discussion

### Conclusions

This study aims to investigate and evaluate women’s experience and engagement with the TreC Mamma app and the virtual coach dedicated to the mindfulness intervention (Maia) and also to assess the level of psychological well-being before and after the intervention.

We expect to collect relevant feedback and suggestions from the participants to possibly improve the structure of the intervention, making it more engaging and improving the interaction. Furthermore, we expect to find differences in terms of improved pre-post psychological well-being, although we are aware that this change may not be related to the intervention itself and that the evaluation of effectiveness will be carried out through a randomized clinical trial at a later stage.

All results will be reported and adequately discussed, including through a comparison with the relevant literature.

### Limitations

This study has several limitations that need to be considered and addressed in future research implementations. First, it is important to consider that this study does not aim to evaluate the effectiveness of the intervention, which is why a control group is not planned. It will be crucial to involve a control group to evaluate the actual effectiveness in a randomized clinical trial study. In addition, this study involves a number of pregnant women from the PAT in Italy. It would be interesting to be able to extend the study to women from other Italian regions as well.

On the one hand, it is important to emphasize that Maia might be a valuable technological support in providing regular psychoeducational services to women during pregnancy.

On the other hand, literature has shown that women during the perinatal period indicated a preference for the support provided on the web, suggesting that the implementation of digital interventions can overcome barriers to social stigma and seeking help. Finally, it is crucial to remember that this technological support (Maia and TreC Mamma) is not a substitute for clinical and medical pathways and life medical support; instead, it is an additional element in promoting psychological well-being and healthy lifestyles during pregnancy.

Should the results of this study be positive, we expect that, after evaluation of effectiveness, the intervention could be made available as a tool to support the psychological well-being of pregnant women.

## Abbreviations

APSS: Azienda Provinciale per i Servizi Sanitari di Trento
FBK: Fondazione Bruno Kessler–Trento
MARS: Mobile Application Rating Scale
NA: negative affection
PA: positive affection
PAT: autonomous province of Trento
QoL: quality of life
UE: user engagement
UES-SF: User Engagement Scale–Short Form
UX: user experience

## Data Availability

The data sets generated during and analyzed during the study are available from the corresponding author on reasonable request.
